# An age-dependent outcome analysis of microvascular decompression and percutaneous thermocoagulation in trigeminal neuralgia

**DOI:** 10.1186/s12883-021-02197-6

**Published:** 2021-04-29

**Authors:** Naureen Keric, Darius Kalasauskas, Sophia L. Kreth, Martin B. Glaser, Harald Krenzlin, Sven R. Kantelhardt, Florian Ringel

**Affiliations:** Department of Neurosurgery, University Medical Center, Johannes Gutenberg University of Mainz, Langenbeckstr. 1, 55131 Mainz, Germany

**Keywords:** Trigeminal neuralgia, Microvascular decompression, Percutaneous thermocoagulation, Old

## Abstract

**Background:**

Trigeminal neuralgia (TN) is a severe pain condition and the most common facial neuralgia. While microvascular decompression (MVD) presents an excellent treatment in neurovascular compression cases, percutaneous thermocoagulation (PT) of the ganglion Gasseri is an alternative option. This study aimed to evaluate post-operative complication rate and outcome of both treatment strategies related to the patient’s age.

**Methods:**

The medical records of all patients with the diagnosis of trigeminal neuralgia undergoing an MVD or PT of the ganglion Gasseri (between January 2007 and September 2017) were reviewed to determine the efficacy and the complication rate of both methods in regard to the patient’s age.

**Results:**

Seventy-nine patients underwent MVD surgery and 39 a PT. The mean age of patients in the MVD group was 61 years and 73 years in the PT group. There were 59 (50%) female patients. Nerve-vessel conflict could be identified in 78 (98.7%) MVD and 17 (43.6%) PT patients on preoperative MRI. Charlson comorbidity index was significantly higher in PT group (2.4 (1.8) versus 3.8 (1.8) *p* < 0.001). The Barrow pain score (BPS) at the last follow-up demonstrated higher scores after PT (*p* = 0.007). The complication rate was markedly higher in PT group, mostly due to the facial hypesthesia (84.6% versus 27.8%; *p* < 0.001). Mean symptom-free survival was significantly shorter in the PT group (9 vs. 26 months, *p* < 0.001). It remained statistically significant when stratified into age groups: (65 years and older: 9 vs. 18 months, *p* = 0.001).

Duration of symptoms (OR 1.005, 95% CI 1.000–1.010), primary procedure (OR 6.198, 95% CI 2.650–14.496), patient age (OR 1.033, 95% CI 1.002–1.066), and postoperative complication rate (OR 2.777, 95% CI 1.309–5.890) were associated with treatment failure.

**Conclusion:**

In this patient series, the MVD is confirmed to be an excellent treatment option independent of patient’s age. However, while PT is an effective procedure, time to pain recurrence is shorter, and the favorable outcome (BPS 1 and 2) rate is lower compared to MVD. Hence MVD should be the preferred treatment and PT should remain an alternative in very selected cases when latter is not possible but not in the elderly patient per se*.*

## Background

Classic trigeminal neuralgia (TN) is defined as a brief, stabbing, and recurrent pain with paroxysmal occurrence in the distribution of one or more branches of the trigeminal nerve without an underlying cause, such as a tumor, an inflammatory or ischemic lesion. It occurs in 12–29/100,000 people annually [3, 12, 22, 26]. The main mechanism of the pain is a demyelination and remyelination process due to vascular pulsatile pressure on the nerve from a so called neurovascular conflict, whereby Aß-fibers trigger Aδ-fibers by ephaptic stimulation [16, 22].

Medical treatment with carbamazepine is the first-line option [9, 22, 32]. However, side effects are common and patients may become refractory to pharmacological treatment [13, 19]. A high rate of patients experience treatment failure in the first 10 years [9]. After failure of conservative treatment or development of medication side effects, patients may switch to invasive treatments. Percutaneous procedures like thermocoagulation, glycerol rhizotomy, or balloon compression of the Gasserian ganglion are less invasive options [21, 36]. Pain relief is achieved by targeted injury of the pain fibers of the trigeminal nerve. Percutaneous thermocoagulation (PT) is a quick procedure, compared to balloon compression or glycerol rhizotomy, resulting in early pain relief [38]. Another safe and efficacious treatment option is radiosurgery of the trigeminal nerve in the prepontine cistern [27]. However, for most cases, especially with clear vascular compression of the trigeminal nerve on high-resolution T2 weighted MR images and refractory pain, microsurgical neurovascular decompression is the treatment of the first choice after failure of conservative treatment [3, 26]. It has proven to be a safe and highly effective treatment [2]. But because of its invasiveness, requiring a craniotomy, it tends to be less commonly performed in older patients whose physicians are prone to continue conservative treatment or repeat percutaneous procedures despite insufficient outcomes.

The Western population is aging. Although biological age does not necessarily correspond to the actual age, older patients often require special precautions. Frailty scores have been established to evaluate their health status, as has the Charlson comorbidity index, a validated tool to predict the probability and length of survival [7]. Even with the application of these tools, it remains unclear whether percutaneous procedures should be the first choice in cases involving older or fragile patients. Therefore, we aimed to analyze the outcomes of the most frequent surgical procedures, percutaneous thermocoagulation, and microvascular decompression, according to patients’ age.

## Methods

### Patient sample and study design

The medical records and radiographs of 118 consecutive patients undergoing PT or microvascular decompression (MVD) for a classic trigeminal neuralgia at our neurosurgical department from January 2007 till October 2017 were retrospectively reviewed. The patient data were de-identified before analysis.

Patients with symptomatic trigeminal neuralgia (most commonly from multiple sclerosis) were excluded. Demographic, clinical, and diagnostic data, including sex, age, patient history, Charlson comorbidity index [7] were assessed. MRI findings of a potential neurovascular conflict, and the perioperative course were collected. The surgical outcome was determined by neurological status, Barrow pain score (BPS) [27], and re-treatment rate at last follow up. Symptom-free survival was measured as the time from the first operation to the recurrence of symptoms if the patient reported at least BPS 3 or if he/she underwent a second operation.

The BPS was recorded as follows:

- BPS 1 – no pain, no medication
- BPS 2 – occasional pain, no medication
- BPS 3 – some pain controlled by medication
- BPS 4 – some pain not controlled by medication
- BPS 5 – severe pain not controlled by medication

### Surgical technique: microvascular decompression

All patients undergoing MVD were operate under general anesthesia via a retrosigmoidal approach using microsurgical technique. In cases involving a clear neurovascular conflict, either Teflon or muscle was used to separate the trigeminal nerve from the vessel.

### Surgical technique: percutaneous thermocoagulation

All PT procedures were performed while the patients were under conscious sedation with additional local anesthesia. Under fluoroscopic control, a thermocoagulation needle was placed 2.5 cm lateral to the mouth angle toward the Gasserian ganglion. After confirmation of the correct position by the patient’s paresthesia response, the nociceptive fibers of the trigeminal nerves were heat injured at 80 degrees Celsius for a duration of 120 s.

### Statistical analysis

The potential risk factors for treatment failure were evaluated using univariate analysis followed by multivariate logistic analysis. The factors studied were age, sex, Charlson comorbidity index, severity of pain, type of procedure, complications, and time from the occurrence of symptoms to the surgical procedure. Categorical data were described by absolute and relative frequencies and continuous data were described by mean, standard deviation, and range. A chi-squared test was used to evaluate the equality of distribution among groups. A Student t-test and Mann-Whitney test were used to compare means between study groups. Comparison of symptom-free survival between study groups was performed using a log-rank test and described using Kaplan-Meier plots. A *p*-value less than 0.05 was considered statistically significant.

## Results

### Patient characteristics

**Fig. 1:**
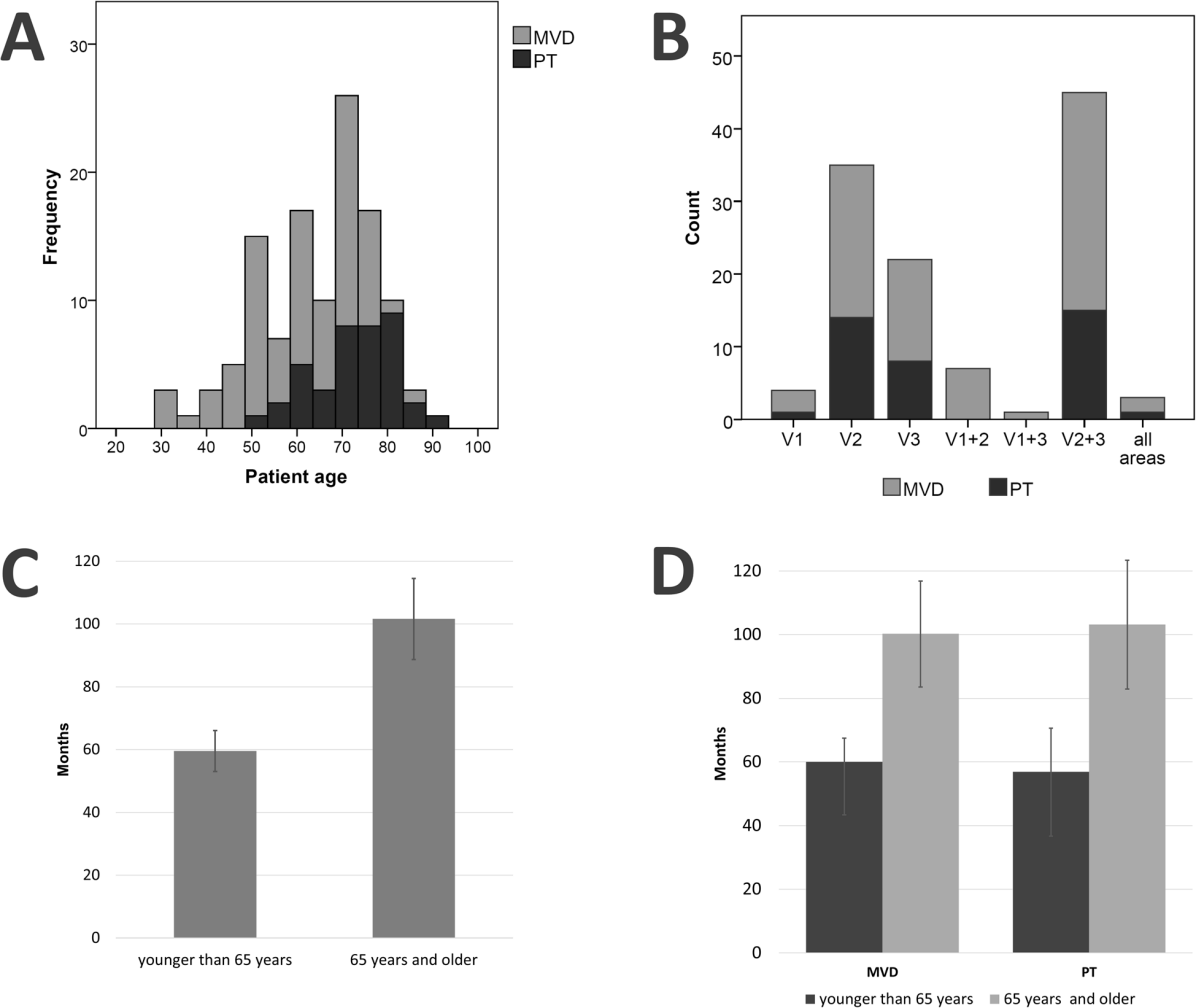
**a** Age distribution of study population based on primary procedure; **b** Distribution of affected trigeminal branches and applied treatment; **c** Comparison of the duration of symptoms before undergoing surgical procedure. Patients are categorized according to their age into younger than 65 years group and 65 years and older group (*p* < 0.001); **d** Comparison of the duration of symptoms before surgical procedure. Patients are categorized based on the primary procedure and their age. No significant difference in time to procedure (either MVD or PT) within age groups

A total of 118 patients with classic TN were included in this study. Between January 2007 and October 2017, 118 patients underwent either MVD or PT of the trigeminal nerve because of conservative treatment failure; 79 patients underwent MVD, and 39 underwent PT. A preoperative MRI was obtained in all MVD cases for diagnostic purposes. The mean age of all patients (MVD + PT) was 64.9 years (range, 31–90 years), and 50% of 118 patients were females (Fig. 1a). The PT group was significantly older in comparison to the MVD group (73 ± 9.6 years (range 49–90 years) and 61 ± 12.2 years (range 31–84 years) (*p* < 0.001); 43% of the patients in the MVD group and 76.9% patients in the PT group were older than 65 years (*p* = 0.001).

The Charlson comorbidity index was significantly higher in the PT group (2.4 ± 1.8 versus 3.8 ± 1.8; *p* < 0.001), this might be an involuntary result of more elderly patients in the PT group. While the cohort presented a spectrum of different pain distribution areas, the second branch of the trigeminal nerve was predominant (V1: 4 (3.4%); V2: 36 (30.5%); V3: 22 (18.6%); V1 + V2: 7 (5.9%); V2 + V3: 45 (37.3%); V1 + V3: 1 (0.8%); V1 + V2 + V3: 3 (2.5%)) (Fig. 1b). The right side was slightly more affected than the left side (right: 64 (54.2%); left: 54 (45.8%)). There was no difference in the pain distribution between the MVD and PT groups. The most frequent reason for surgical treatment was conservative treatment failure (59 (74.7%) in the MVD and 30 (76.9%) in the PT group). The rest of the patients indicated side effects from medication and insufficient pain relief as the reason for surgical treatment. A neurovascular conflict was identified on high resolution T2-weighted MR images in 98.7% of patients in the MVD group and 43.6% of patients in the PT group. Vascular compression of the trigeminal nerve was predominantly caused by the superior cerebellar artery (SCA; 63.4%), followed by the anterior inferior cerebellar artery (AICA; 12.7%); in 23.9%, the artery was not clearly defined, and in 16.8% of cases a vein was found intraoperatively to be in conflict with the trigeminal nerve. Charlson comorbidity index in the PT group was 2.41 ± 1.8 in MVD and 3.85 ± 1.8 in the PT group (*p* < 0.001). Patient characteristics are provided in Table 1.

**Table 1:** Clinical characteristics of the study sample

Characteristics	Total	MVD	PT
No of patients	118	79	39
Mean age	64.9 (31–90)	60.9 (31–84)	72.7 (49–90)
**No of patients in age groups**			
< 65 years	54	45	9
> 65 years	64	34	30
**Sex**			
F	59	39	20
M	59	40	19
**Facial side**			
Left	54	33	21
Right	64	46	18
**Trigeminal branch**			
V_1_	4	3	1
V_2_	36	21	15
V_3_	22	14	8
V_1_ + V_2_	7	7	0
V_1_ + V_3_	1	1	0
V_2_ + V_3_	45	31	14
V_1_ + V_2_ + V_3_	3	2	1
**No of treatments**			
Total	155	87	68
No of treatments per patient	1.3	1.1	1.7
**Preoperative mean duration of symptoms ± SD:**			
Total	82.6 (±84.4)	77.5 (±75.1)	92.5 (±100.6)
< 65 years	59.5 (±47.2)	60.0 (±48.8)	56.9 (±41)
> 65 years	101.6 (±102.3)	100.2 (±95.6)	103.2 (±110.8)
**Mean follow-up (months) ± SD**	12.1 (±18.8)	11.7 (±21.0)	12.7 (±13.7)
**Indication for surgical treatment**			
Conservative treatment failure	89	59	30
Medical side effects	11	9	3
Combination of both	18	12	6
**Pain recurrence rate (%)**			
Total	51 (43.2)	23 (29.1)	28 (71.8)
< 65 years	19 (35.2)	11 (24.4)	8 (88.9)
> 65 years	32 (50.0)	12 (35.3)	20 (66.7)
**Mean time to re-surgery (months) ± SD**			
Total	11.7 (±10.5)	12.9 (±9.0)	10.8 (±11.6)
< 65 years	13.6 (±11.6)	14.3 (±12.1)	12.8 (±12.3)
> 65 years	10.7 (±9.9)	11.9 (±6.5)	10 (±11.6)
**Charlson comorbidity index ± SD**	2.9 (±1.9)	2.4 (±1.8)	3.8 (±1.8)

### Time to surgery; surgical treatment; and complication rates

Analysis of the time to treatment for the entire patient cohort clearly showed that the duration of symptoms before undergoing surgical treatment was much longer in older than in younger patients (> 65 years; 101.6 ± 102.3 months versus < 65 years; 59.5 ± 47.2 months; *p* = 0.004) (Fig. 1c). The difference remained after dividing the cohort into MVD and PT groups. In the MVD group, the duration of symptoms in patients older than 65 years was 100.2 ± 95.6 months, and in the younger patients it was 60 ± 48.8 months (*p* = 0.03) (Fig. 1d). In the PT group, the symptom duration in older patients was 103.2 ± 110.8 months and in the younger patients it was 56.9 ± 41.0 months (*p* = 0.06). In total, 79 patients underwent 87 MVD procedures (1.1 procedures per patient) and 39 patients underwent 68 PT procedures (1.7 procedures per patient).

A total of 55 patients experienced a postoperative complication (22 in the MVD group and 33 in the PT group). Facial hemihypesthesia was the most frequent complication in both groups: 79.5% of patients in the PT group and 17.7% of patients in the MVD group experienced this complication. No case of painful facial or corneal anesthesia was observed in both treatment groups. Other complications included loss or deterioration of hearing in two patients (one in each group), CSF fistula and wound healing problems in five patients in the MVD group (6.3%), and pneumonia or pulmonary embolism (one in each group). One patient died due to fulminant pulmonary embolism in the MVD group. The proportion of patients who developed complications was similar in both age groups (> 65 years: 60.9% versus < 65 years: 29.6%; *p* = 0.001) (Table 2).

**Table 2:** Complications

	Total	MVD	PT
**No of patients with complications**	55 (46.6%)	22 (27.8%)	33 (84.6%))
< 65 years	16 (29.6%)	9 (20.0%)	7 (77.8%)
> 65 years	39 (60.9%)	13 (38.2%)	26 (86.7%)
**Facial hypesthesia**			
< 65 years		7	7
> 65 years		7	24
**CSF fistula**			
< 65 years		1	
> 65 years		3	
**Hearing loss**			
< 65 years			
> 65 years		2	2
**Death**			
< 65 years			
> 65 years		1	
**Other**			
< 65 years		3^a^	
> 65 years		4^b^	3^c^

^a^ include mild trochlear paresis and dysgeusia

^b^ include hemiparesis after stroke on the 2nd postoperative day, dysgeusia and wound seroma

^c^ include AV block II°

### Outcomes

**Fig. 2:**
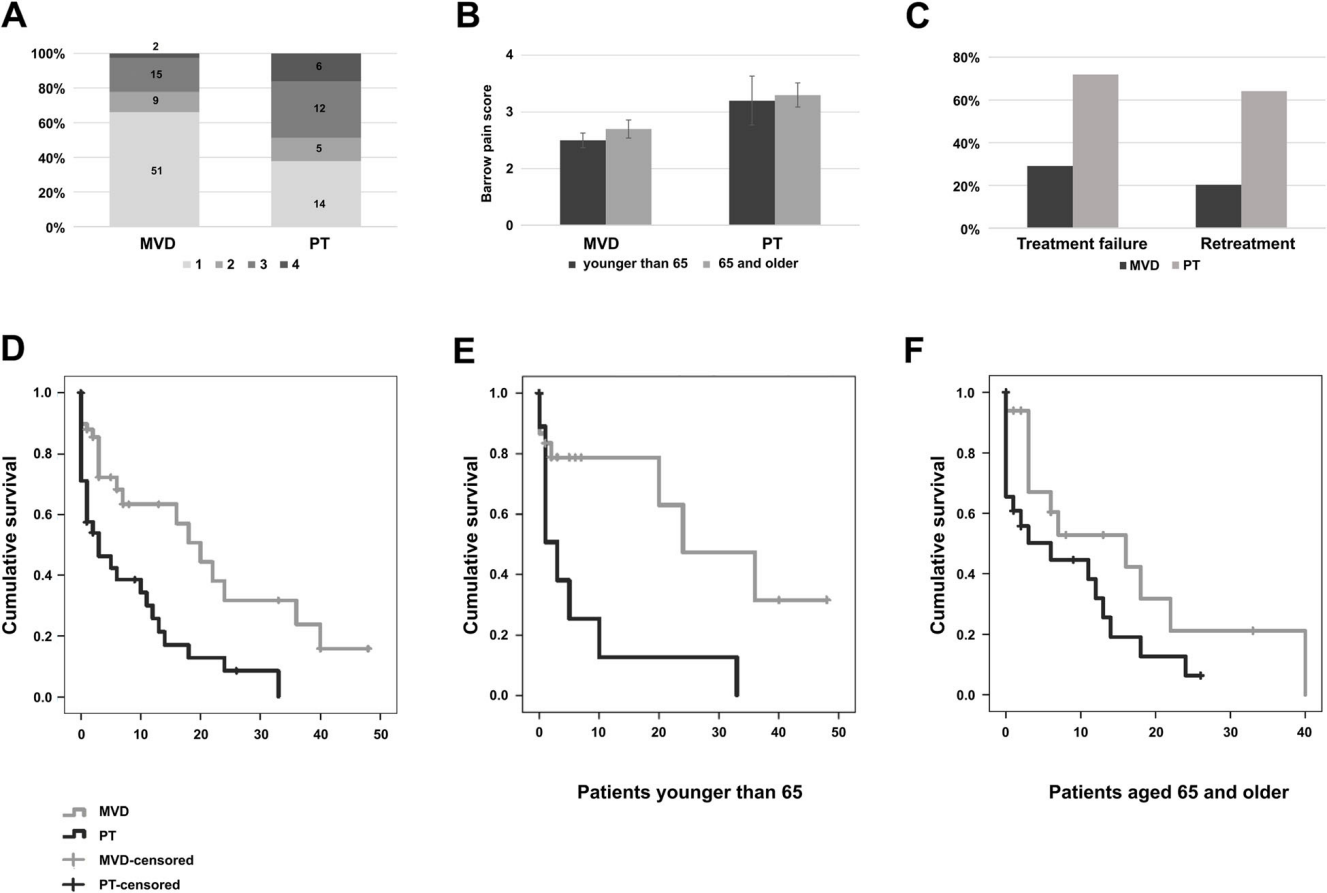
**a** The distribution of the level of pain after the procedure according to BPS, *p* = 0.007; **b** The level of pain after the procedure according to BPS stratified by age; **c** Distribution of treatment failure and retreatment based on primary surgical procedure. In 51 (43.2%) patients, the surgical treatment was unsatisfactory, i.e. they have undergone another surgical procedure or reported a pain equivalent to BPS 3 or more (*p* < 0.001 for both); **d** Survival until the second procedure is performed or the pain after the procedure reaches BPS 3 **e** Time to re-treatment for patients younger than 65 years (*p* < 0.001); **c** Time to re-treatment for patients 65 years and older (*p* < 0.001)

Outcomes were measured by the BPS. The mean BPS score after MVD for patients younger than 65 years was 1.5 ± 0.9 and 1.7 ± 0.9 for those older than 65 years (*p* = 0.52). In the PT group, the mean BPS score for the younger patients was 2.2 ± 1.3 and was 2.3 ± 1.1 for the older patients (*p* = 0.88). No patient in the MVD group had a BPS of 4 and 5 (Fig. 2 a, b).

Pain recurrence was defined as BPS 3 or the timepoint for indication for re-treatment. In the comparison of the elderly patients in both treatment groups, a significantly higher BPS was found in the PT group (*p* = 0.02). As a consequence of more elderly patients in the PT group, the recurrence rate was much higher in this group, so that the influence of age on pain recurrence was analyzed. No difference in time to recurrence was found among the younger and older patients in either the MVD or the PT group (MVD: 16.1 ± 4.1 months in > 65 versus 27.9 ± 4.8 months in < 65 *p* = 0.45; PT: 8.2 ± 1.9 months in > 65 versus 6.9 ± 4 months in < 65 *p* = 0.86). Regardless, the time to recurrence was significantly shorter in the PT group compared to the MVD group (8 ± 1.9 versus 26 ± 3.4 months, *p* < 0.001).

Treatment failure occurred more often in the PT group compared to the MVD group (69.2% versus 27.8%; *p* = 0.001). Furthermore, re-treatment was similarly distributed (61.5% in the PT group versus 20.3% in the MVD group; *p* < 0.001). When comparing these outcomes among the younger and older patients, we found a trend towards higher incidence of treatment failure in the older group (25.9% in the < 65 group versus 40.6% in the > 65 group; *p* = 0.12). This might result from the greater distribution of older patients in the PT group (Fig. 2 c-f). In 40 patients (33.9%), a second procedure was necessary. A second MVD was performed in 6 patients (7.6% of MVD cohort), a second PT in 19 (47.53% of PT cohort) patients. Further, 10 (12.7%) patients received PT after unsatisfactory MVD (BPS 3) and 5 (12.8%) MVD after a PT. Two patients were treated with motor cortex stimulation after PT and one with radiosurgery prior to PT. There was a significantly larger proportion of retreatment in PT group (20.3 vs. 61.5%, *p* < 0.001).

To find influencing factors for pain recurrence, univariate regression analysis was performed using the following factors: age, duration of symptoms, primary procedure, complications, and the Charlson comorbidity index. We found that the duration of symptoms OR 1.005, 95% CI 1.000–1.010) is the most pertinent factor for pain recurrence, followed by primary procedure (OR 6.198, 95% CI 2.650–14.496), patient age (OR 1.033, 95% CI 1.002–1.066), and postoperative complication (OR 2.777, 95% CI 1.309–5.890). Charlson comorbidity index and sex were statistically not significant. Both the primary procedure (OR 6.293, 95%CI 2.622–15.103) and the duration of symptoms (OR 1.005, 95%CI 1.000–1.011) retained their statistical significance in a multivariate analysis.

## Discussion

Since the introduction and establishment of the MVD procedure by Peter J. Jannetta in 1967 [18], the method has been refined and applied to other neurovascular conflict syndromes such as hemifacial spasm [10, 31]. PT is a well-established alternative method for older patients or patients with comorbidities [36]. Here we assessed the safety and efficacy of both procedures according to patient age. We found an age-independent favorable outcome after MVD compared to PT. The postoperative pain intensity measured by BPS, as well as the complication rate, were significantly lower after MVD. Moreover, the time to recurrence was shorter in PT patients.

The characteristics of our patients largely correspond to those described in published reports involving larger patient cohort [2, 5, 8, 31, 34]. Classic TN is a disease of older age; accordingly, the mean age of our patient cohort was 64.9 years.

### Treatment and complication rates

On average, 7.3 years elapsed before the first surgical treatment in our patients overall. Among the patients who underwent MVD, the average symptom duration prior to the MVD procedure was 6.7 years. In the study reported by Broggi et al. [5], the duration of the symptoms up to the time of the first surgical intervention was approximately 8.5 years; in that reported by Barker et al., it was 6 years [2].

Numerous studies have examined whether the duration of symptoms before the first intervention has a negative impact on the success of the operation [2, 5, 20]. This theory assumes that long compression of the nerve could lead to degeneration of nerve fibers [44]. Some authors examined this relationship and found a significant negative correlation between preoperative symptom duration and long-term surgical success [2, 5, 39]. Our results accord with this finding. In our cohort, the duration of symptoms was a significant factor influencing treatment success. In contrast, Sindou et al. and Sun et al. did not identify a statistically significant connection between symptom duration and time to recurrence [34, 35].

In a study reported by Miller et al., patients with classic TN were divided into two groups based on the characteristics of their pain symptoms: Group 1 patients had pain attacks with pain-free intervals more than 50% of the time; group 2 patients had persistent pain symptoms more than 50% of the time [23]. It was observed that some of the patients with long-term seizure-like pain symptoms subsequently developed constant pain (those experienced more than 50% of the time) [20, 23]. This observation supports the current leading hypothesis on the pathophysiology of TN and the suspicion that progressive disease results from ongoing nerve compression. Further, Miller et al. found that group 1 patients had better long-term surgical outcomes after MVD than group 2 patients [23]. Szapiro and Li also noticed a trend towards a poorer surgical outcome in patients with constant pain [20, 37]. Given the possibility of a worsening of symptoms from seizure-like to continuous, together with a possibility of a worse prognosis after MVD, consideration should be given to surgical therapy at an early rather than later stage.

Vascular compression effects the degenerative changes and the resulting elongation of the vessels with increasing age [16]. The most common compression diagnosed intraoperatively is caused by the superior cerebellar artery (75%); 10% of the compressions are caused by the anterior inferior cerebellar artery. Venous compression is found in 12% of cases, either individually or more commonly (in 68% of cases involving venous compression) in combination with an artery [2, 32]. In our study, the distribution of the vessels involved was similar to that reported previously. Venous compression was found in 16.8% of patients. A negative effect on long-term postoperative freedom from pain caused by venous compression has been described previously [2, 20, 34]. In our study, 7 of the patients with venous compression had an operative outcome of BPS 2 or 3, while 13 patients had an excellent postoperative result.

Postoperative complications occurred in 27.8% of the patients who underwent decompression. In contrast, the complication rate in patients who underwent PT was 82.1%. The most common complication of PT was postoperative hypesthesia in the affected innervation area; 32 of 39 patients who underwent PT experienced this complication. Excluding postoperative hypesthesia, the complication rate in patients who underwent PT was 10%; that of the patients who underwent decompression was 10.1%.

Postoperative sensitivity disorder after PT is also described in the literature as the most common complication, occurring in 80% of cases when the temperature exceeded 70 °C or in 81% of cases in general [30, 43]. Postoperative hypesthesia in the trigeminal nerve distribution is an unavoidable side effect if PT is performed correctly. Excluding sensory loss from the complication rate, Lopez reports postoperative complications in 29.2% of cases [21].

Hearing loss after PT occurred in two patients. The incidence of hearing loss in our patients (2.5%) is lower than in previous studies [11, 14]. Postoperative cerebrospinal fluid (CSF) fistula occurred in four patients in the MVD group, whereby none of them developed postoperative meningitis. Sensitivity disorders and CSF fistulas occurred comparatively more frequently in our patients than in other previously reported cohorts. Second-degree AV block occurred in two patients. Two patients reported postoperative dysgeusia.

One patient died after MVD due to postoperative pulmonary embolism, that did not resolve despite emergency and intensive care unit treatment. The reported average mortality rate after MVD is 0.2–0.5% [2, 14, 28]. The mortality rate in our study, with one patient death in 79 patients who underwent MVD (1.2%), is slightly higher.

### Outcomes

According to the surgical method, the outcomes were as follows: Of the patients who underwent MVD, 82.4% showed a very good or good response to surgery (BPS 1 and 2). In contrast, only 42.2% of the patients who underwent PT had a good or very good response to surgery. The time at which the surgical outcome was recorded refers to the last follow-up time, which occurred on average in the MVD patients after 11.7 months and in the PT patients after 12.7 months. These outcomes support the view of many previous investigators that MVD is the most effective treatment option for patients with classic TN in terms of pain reduction [40].

The definition of therapeutic success and the determined success rates for MVD vary in the literature. Their extensive study involving 1155 MVD patients followed for 1 year or longer after the operation, Barker et al. determined success rates of 80% after 1 year and 74% after 10 years. They defined recurrence or treatment failure correlating to BPS grade 4 [2]. In our cohort, 16.2% of the patients in the MVD group had a BPS score of 3 postoperatively, namely, recurring pain symptoms that could be controlled with medication.

Among our patients who underwent PT, 30 (75%) suffered a pain recurrence, which was treated conservatively in some and through further surgical interventions in others. This comparatively high recurrence rate can be explained by the fact that in comparison to the literature, no uniform time for a follow-up was defined to assess a potential recurrence. It should also be kept in mind that no uniform coagulation strength and duration have been determined, and study results may, therefore, vary [17].

The recurrence rate after MVD in our study was 31.6% (25 patients) with a mean follow-up period of 11.7 months. Of the patients with pain recurrence, 18.9% (15 patients) reported a BPS score of 3 (pain symptoms controlled with medication). Relapse rates of 24 and 21.4% have been previously reported [2, 41]. The pain relapse occurred in our MVD patients after an average of 9.4 months. This corresponds to previously reported data showing a recurrence of pain frequently recorded in the first 2 years after the operation [2]. After 2 years, the risk of recurrence is low, and relapse may be due to an early dislocation of the teflon patch [2].

### Age

Since classic TN occurs increasingly with age, the risks of surgical treatment of the disease in older people have become the subject of research. Several retrospective studies [1, 15, 24, 29] and a prospective study examined the influence of age on the surgical treatment outcome [33]. The studies included relatively small patient populations and investigated the appearance of complications in older patients undergoing MVD. The results indicated that older patients are not at increased risk of a severe complication of MVD (cerebral hemorrhage, stroke, cranial nerve injury, or death). This assumption was confirmed in Sekula’s meta-analysis [33]. Rughani evaluated mortality in a cohort of MVD patients and found an age-independent mortality rate of less than 0.5% [28].

In our cohort, one patient died after MVD surgery due to postoperative pulmonary embolism; the patient’s age at the time of surgery was 72 years. Analysis of the complication rates in our patient population shows that in both the MVD and PT groups, the complication rates increased with age. This might be an effect of the older patient population in the PT group, which showed a high complication rate caused by postoperative hypesthesia.

No increase in complications in elderly patients after MVD was observed [29]. Broggi et al. consider older age as a favorable factor for MVD due to cerebellar atrophy affording easier access to the operating area [5]. In another study, a higher complication rate in the group of older patients was seen [4]. The complication rates in our MVD group increased with age but remained lower than the complication rates in older patients in the PT group. Rughani described a relationship between the patient’s age and cardiac, pulmonary, and cerebrovascular complications [28]. It is not recommended to use the criterion age as a crucial factor [6]. Rughani advocates that patient selection should rather be based on an individual patient’s risk profile. Although age can serve as a criterion for estimating the risk of surgery, as it correlates with other variables such as comorbidities. Therefore, we measured the Charlson comorbidity index as a potential influencing factor. However, its influence could not be verified in our patient cohort.

From our collected data and the statistical analysis, we found that the likelihood of success with regard to very good pain control (BPS 1) was worse for PT than for MVD. However, the prevalent conclusion in the literature is that there is no difference in the success of surgery in younger and older patients [15, 25, 33, 45].

In our comparison of the success rates of the two surgical methods (PT and MVD) according to patient age, we found that MVD also results in better surgical outcomes in older patients than PT. In the MVD group, good surgical outcome decreases and the recurrence rate increases with increasing age. Similar trends were seen in the PT group. In contrast, Ogungbo found that the recurrence rate in MVD patients decreases with age [24]. Gunther, on the other hand, saw no significant difference in the rate of recurrence in younger and older patients; the recurrence rate was 20.4% in the patients older than 64 years and 13.4% in those aged 64 years and younger, and it was highest in the first 12 months postoperatively [15]. Yang also found no difference in the relapse rate depending on the age of the patient. When considering the time to pain recurrence after MVD, there was a significantly longer duration of pain relief in young patients [42]. Similarly, Ashkan described a considerably shorter time to pain recurrence in MVD patients older than 60 years [1].

The longest preoperative symptom duration before surgical treatment in our survey was found among the patients older than 65 years, with an average symptom duration of 120.8 months. Therefore, the significantly shorter time to relapse in older patients is attributed to irreversible nerve damage with long preoperative symptom duration. Nevertheless, overall, the mean duration of pain relief in the MVD group was longer ​​than in the PT group in our study [1, 5].

## Conclusion

Surgical therapies for the treatment of classic TN are indicated, when drug therapy for pain control is no longer sufficient, or the side effects are no longer tolerable for the patient. MVD is performed primarily in relatively young patients in good general health. PT is performed in addition to or instead of MVD in patients in whom MVD is considered dangerous or otherwise not possible. The results of this study confirm that even with increasing patient age, MVD achieves better surgical outcomes than PT and is not associated with a complication rate similar to PT. MVD in patients with classic TN therefore should remain the primary surgical option even in the elderly population. The patient’s age should not be used as an isolated risk factor, but rather, as a co-factor for predicting potential comorbidities.

## Data Availability

No data from public databases has been used in this study. No administrative permissions were required to access patient data.

## Abbreviations

AICA: Anterior inferior cerebellar artery
BPS: Barrow pain score
CI: Confidence interval
CSF: Cerebrospinal fluid
MRI: Magnetic resonance imaging
MVD: Microvascular decompression
OR: Odds ratio
PT: Percutaneous thermocoagulation
SCA: Superior cerebellar artery
TN: Trigeminal neuralgia
